# Epigenetic Reprogramming of Tumor-Associated Fibroblasts in Lung Cancer: Therapeutic Opportunities

**DOI:** 10.3390/cancers13153782

**Published:** 2021-07-27

**Authors:** Jordi Alcaraz, Rafael Ikemori, Alejandro Llorente, Natalia Díaz-Valdivia, Noemí Reguart, Miguel Vizoso

**Affiliations:** 1 Unit of Biophysics and Bioengineering, Department of Biomedicine, School of Medicine and Health Sciences, Universitat de Barcelona, 08036 Barcelona, Spain; rafaelikemori@ub.edu (R.I.); allorente@ub.edu (A.L.); ndiazvaldivia@ub.edu (N.D.-V.); 2 Thoracic Oncology Unit, Hospital Clinic Barcelona, 08036 Barcelona, Spain; NREGUART@clinic.cat; 3 Institute for Bioengineering of Catalonia (IBEC), The Barcelona Institute for Science and Technology (BIST), 08028 Barcelona, Spain; 4 Institut d’Investigacions Biomèdiques August Pi i Sunyer (IDIBAPS), 08036 Barcelona, Spain; 5 Division of Molecular Pathology, Oncode Institute, The Netherlands Cancer Institute, Plesmanlaan 121, 1066 CX Amsterdam, The Netherlands

**Keywords:** lung cancer, cancer-associated fibroblasts, epigenetics, desmoplasia, TGF-β, smoking, tumor stroma

## Abstract

**Simple Summary:**

Lung cancer is the leading cause of cancer death among both men and women, partly due to limited therapy responses. New avenues of knowledge are indicating that lung cancer cells do not form a tumor in isolation but rather obtain essential support from their surrounding host tissue rich in altered fibroblasts. Notably, there is growing evidence that tumor progression and even the current limited responses to therapies could be prevented by rescuing the normal behavior of fibroblasts, which are critical housekeepers of normal tissue function. For this purpose, it is key to improve our understanding of the molecular mechanisms driving the pathologic alterations of fibroblasts in cancer. This work provides a comprehensive review of the main molecular mechanisms involved in fibroblast transformation based on epigenetic reprogramming, and summarizes emerging therapeutic approaches to prevent or overcome the pathologic effects of tumor-associated fibroblasts.

**Abstract:**

Lung cancer is the leading cause of cancer-related death worldwide. The desmoplastic stroma of lung cancer and other solid tumors is rich in tumor-associated fibroblasts (TAFs) exhibiting an activated/myofibroblast-like phenotype. There is growing awareness that TAFs support key steps of tumor progression and are epigenetically reprogrammed compared to healthy fibroblasts. Although the mechanisms underlying such epigenetic reprogramming are incompletely understood, there is increasing evidence that they involve interactions with either cancer cells, pro-fibrotic cytokines such as TGF-β, the stiffening of the surrounding extracellular matrix, smoking cigarette particles and other environmental cues. These aberrant interactions elicit a global DNA hypomethylation and a selective transcriptional repression through hypermethylation of the TGF-β transcription factor *SMAD3* in lung TAFs. Likewise, similar DNA methylation changes have been reported in TAFs from other cancer types, as well as histone core modifications and altered microRNA expression. In this review we summarize the evidence of the epigenetic reprogramming of TAFs, how this reprogramming contributes to the acquisition and maintenance of a tumor-promoting phenotype, and how it provides novel venues for therapeutic intervention, with a special focus on lung TAFs.

## 1. Introduction

Lung cancer is the leading cause of cancer-related deaths worldwide, and smoking and aging are major risk factors [1,2]. Histologically, non-small cell lung cancer (NSCLC) is the most frequent lung cancer subtype, and it is subdivided into adenocarcinoma (ADC, which typically arises in distal pulmonary sites and is common in non-smokers), squamous cell carcinoma (SCC, which is frequently found in proximal airways and is strongly associated with smoking), and other less frequent subtypes [1]. Although all these subtypes are epithelial in origin, it is now clear that the interplay between carcinoma cells and their surrounding desmoplastic stroma is essential for cancer establishment and progression [3,4,5]. 

The most abundant cell types within the tumor stroma of solid tumors are the tumor-associated fibroblasts (TAFs) or cancer-associated fibroblasts (CAFs), a spindle-shaped cell type of mesenchymal lineage [6,7]. Under the pressure of a transformed environment, TAFs are frequently activated, acquiring pro-fibrotic/myofibroblast-like features [8]. Notably, preclinical studies have implicated TAFs in all major steps of tumor progression, including cancer cell growth, invasion, angiogenesis and immunosuppression, through the secretion of tumor-promoting factors [7,9]. Moreover, TAFs are the key effector cell type in the excessive deposition of fibrillar collagens and other pro-fibrotic extracellular matrix (ECM) components that may shield cancer cells from immune checkpoint inhibitors or targeted therapies [10], thereby posing a major barrier against cancer therapeutics in lung cancer and other solid tumors [11]. 

Unlike normal fibroblasts, TAFs can maintain their tumor-promoting phenotype for numerous passages during cell culture in vitro without exposure to cancer cells [12,13], thereby suggesting an underlying intrinsic “memory”. Such “memory” appears to rely on epigenetic modifications rather than genetic alterations, since TAFs are considered genomically stable in terms of copy number variations, chromosomal aberrations and point mutations [14], unlike cancer cells. 

Epigenetic regulatory mechanisms include all stable and long-term chromatin-template processes linked to the transcriptional potential of a cell regardless of the heritability potential. These mechanisms are commonly categorized in three main types: DNA methylation, histone core modifications and non-coding RNAs. Notably, these epigenetic mechanisms provide transcriptional control by regulating chromatin compaction in a highly coordinated manner, eliciting either open (euchromatin) or close chromatin (heterochromatin) regions, which are associated with gene activation or repression, respectively [15]. In the following sections we summarize the evidence supporting the epigenetic reprogramming of TAFs and the potential underlying mechanisms, with special emphasis on lung TAFs. Moreover, we include a final section on the emerging therapeutic opportunities elicited by such epigenetic reprogramming.

## 2. Evidence That Tumor-Associated Fibroblasts (TAFs) Are Epigenetically Reprogrammed

It is now clear that TAFs are molecularly and phenotypically heterogeneous in NSCLC and other solid tumors [16,17]. Yet recent work from our group supports that there is a dominant TAF phenotype in each histologic subtype [18,19,20], which accounts for the differential response to the antifibrotic drug nintedanib reported in TAFs in ADC and SCC in preclinical models [19,21] and validated in the LUME-1 trial in patients [22]. A deeper understanding of the epigenetic regulation of TAF phenotypes may shed light on their stability and potential interconvertibility [23], and may help in developing therapeutic strategies aiming to revert TAFs towards tumor-restraining phenotypes. Below, we summarize recent evidence of major reported epigenetic changes in TAFs.

### 2.1. DNA Methylation Changes in TAFs

The best studied epigenetic modification is DNA methylation, which consists of the deposition of a methyl group (CH_3_) on the C5 position of cytosines (5mC), which occurs largely in clustered DNA regions called CpG islands [24,25,26]. The enzymes responsible for reading, writing and erasing the methylome code are the DNA methyltransferases (DNMTs: the maintenance DNMT DNMT1 and the de novo DNMTs DNMT3A and DNMT3B) and the methylcytosine dioxygenase enzymes (TETs: TET1–3) [27]. 

**Figure 1:**
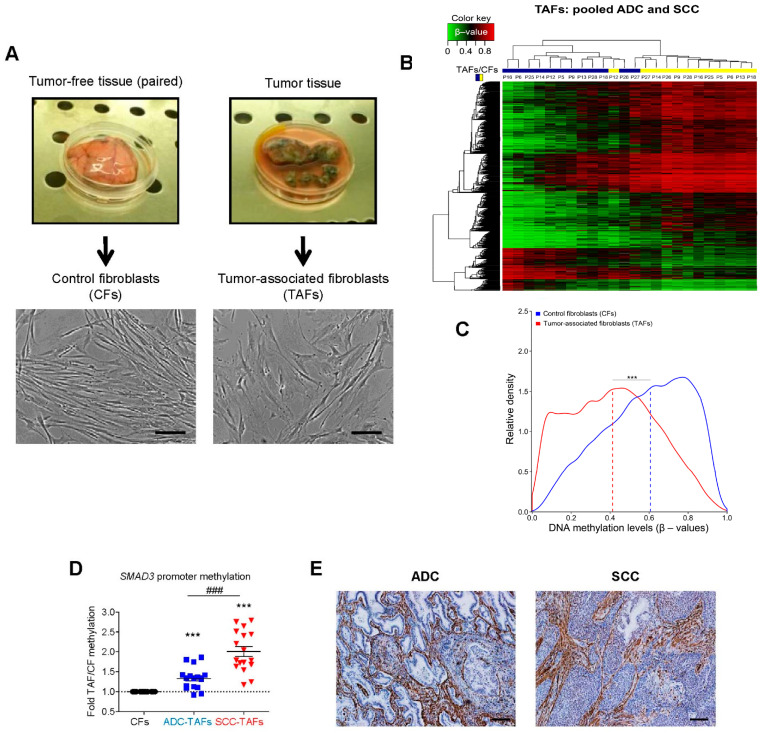
Hallmarks of the epigenetic reprogramming of tumor-associated fibroblasts (TAFs) from lung cancer patients. (**A**) Representative images of a tumor tissue (right) and patient-matched tumor-free pulmonary tissue sample (left) obtained from a surgical lung cancer patient (top row). These tissue samples were used to obtain TAFs and paired control fibroblasts (CFs) following a tissue explant protocol [20]. Scale bar, 50 μm. (**B**) Heatmap corresponding to the unsupervised clustering of the most differentially methylated CpG sites in TAFs (blue) and paired CFs (yellow) obtained from 12 lung cancer patients. Low and high DNA methylation levels (β-values) are shown in green and red, respectively. Data from TAFs from adenocarcinoma (ADC) and squamous cell carcinoma (SCC) patients were combined. Reprinted from [32] with permission. (**C**) Histogram showing the relative density of the average DNA methylation levels across TAFs (red line) and CFs (blue line) shown in (**B**). Vertical dashed lines indicate the median β-values. Reprinted from [32] with permission. *** *p* < 0.001. (**D**) DNA methylation of 3 CpG sites within the *SMAD3* promoter of CFs (*n* = 12). ADC-TAFs (*n* = 6) and SCC-TAFs (*n* = 6) assessed by pyrosequencing. Reprinted from [19] with permission. ### *p* < 0.005, *** *p* < 0.005. (**E**) Representative histologic staining of the standard fibroblast activation marker α-SMA in an ADC and SCC patient. Scale bar, 200 μm.

By mapping the global DNA methylation patterns in TAFs derived from gastric cancer patients, it was discovered that the epigenetic reprogramming of TAFs is characterized by global hypomethylation concomitantly with rare focal gains of methylation, which is qualitatively similar to the epigenetic alterations observed in cancer cells and independent of imbalanced expression of DNMTs [28]. A similar global hypomethylation was found in TAFs in a mouse model of pancreatic carcinoma [29], and in breast tumors compared to normal stroma [30]. In the context of the lung, the first analysis of DNA methylation in TAFs using a low-resolution microarray identified the *LPXN* gene as the single differentially methylated gene in lung TAFs compared to patient matched control fibroblasts (CFs) [31]. Our group subsequently expanded this list up to 750 genes using a higher resolution microarray, reporting a global DNA hypomethylation concomitantly with a selective impact on *SMAD3* and other key transcription factors of the TGF-β pathway (Figure 1A–C) [32]. More recently, we found that the epigenetic repression of *SMAD3* through hypermethylation was significantly larger in SCC-TAFs than ADC-TAFs (Figure 1D), revealing that the epigenetic reprogramming of lung TAFs depends on their histologic subtype, at least in part [19].

The aberrant DNA methylome of lung TAFs may have a global impact on gene expression, since nearly half of the 46 genes exhibiting a marked differential expression between lung TAFs and control fibroblasts (CFs) reported by Navab and coworkers [31] also exhibited differential DNA methylation in our study, including the hypermethylation of the T-Box transcription factor *TBX4* [32]. In agreement with this observation, another study reported the *TBX4* gene and 6 other transcription factors to be downregulated concomitantly with DNA hypermethylation in lung TAFs compared to patient-matched control fibroblasts, including *TBX2*, *FOXL1*, *BACH2*, *SIX4*, *SOX9* and *HOXA5* [33]. Collectively, these studies reveal that global hypomethylation concomitantly with selective hypermethylation of prominent transcription factors such as *SMAD3* is an emerging hallmark of lung TAFs. However, the role of such hypomethylation in the plasticity and tumor-promoting effects of TAFs remains incompletely understood.

### 2.2. Histone Core Modifications in TAFs

The plasticity of the chromatin configuration and DNA 3D-loop formation between adjacent or distant regions can be determined by the post-translational chemical alterations of the histone amino acid residues through methylation, acetylation and other processes [34,35,36]. Acetylation of histone 3 (H3) lysine 9 or 27 (H3K9 or K27) by histone acetyltransferases (HATs) is associated with transcriptional activation, whereas deacetylation by histone deacetylases (HDACs) relates with a close chromatin conformation [36]. To our knowledge, most previous work on histone modifications in TAFs was conducted in the context of fibroblast activation and is summarized in Section 3.2. Interestingly, a recent proteomic analysis of ovarian tumors reported that the metastatic stromal proteome was notably uniform and characterized by the overexpression of the nicotinamide N-methyltransferase (NNMT) as well as several proteins regulated by it. Such uniformity of the stromal proteome was in marked contrast with the genetic and proteomic heterogeneity of the corresponding carcinoma compartment [37]. NNMT is a key metabolic enzyme in catalyzing the N-methylation of nicotinamide (one of the forms of vitamin B3) using the universal methyl donor S-adenosyl methionine (SAM) [38]. Notably, the overexpression of stromal NNMT in ovarian tumors led to the depletion of SAM concomitantly with DNA and histone hypomethylation, which were in turn associated with widespread gene expression alterations. Conversely, NNMT knockdown increased histone methylation at residues associated with transcriptional regulation, including an increase in H3K4 and H3K27 trimethylation. Moreover, it was reported that NNMT expression was necessary and sufficient to elicit a tumor-promoting phenotype in TAFs in vitro and in vivo, revealing that histone methylation has a major role in shaping such a pro-malignant phenotype, and supporting that NNMT or other aberrant methyltransferase activities in TAFs could be a therapeutically interesting target to normalize the tumor stroma [37]. In agreement with this interpretation, NNMT was upregulated in TAFs in gastric and colorectal cancer [39], and is associated with poor prognosis in several solid tumors including NSCLC. Moreover, NNMT overexpression was linked with the expression of PD-L1 and other immune checkpoint markers [38,40], whereas NNMT silencing was associated with decreased tumorigenicity in NSCLC [41]. 

Unlike NNMT, another study reported that inflammation downregulates the histone methyltransferase zeste homolog-2 (EZH2) and specifically demethylates H3K27me3 marks in TAFs from gastric cancer patients, which was essential to drive a senescence-associated secretory phenotype in TAFs that enhanced peritoneal dissemination in a mouse model [42]. Moreover, EZH2 was implicated in TAF-dependent angiogenesis in hepatocellular carcinoma through VEGF overexpression [43]. Collectively, all these studies underscore the tumor-promoting effects of N-methyltransferases such as NNMT and EZH2 in TAFs. Yet it remains to be confirmed if these methyltransferases are aberrantly expressed and/or exhibit tumor-promoting traits in lung TAFs.

### 2.3. Non-Coding RNA Alterations in TAFs

Epigenetic modulators based on non-coding RNAs can be grouped in microRNAs (miRNAs, 20–22 bp), long non-coding RNAs (>200 bp) and exonic/intronic circular RNAs. MicroRNAs are the most well-studied non-coding RNAs and act by tethering to the target messenger RNA to mediate their degradation or translation blockade [44]. MicroRNAs have been implicated in the transformation of CFs into TAFs in a variety of solid tumors including NSCLC [45]. A recent comprehensive review of miRNAs in TAFs in hepatocellular carcinoma revealed that their dysregulation affects numerous biological processes, including transcription factors, ECM and cytoskeletal components, and EMT regulators, and is involved in the aberrant activation of TAFs (further discussed in Section 3). Notably, the most common aberrant miRNA expression in TAFs in numerous solid tumors includes the downregulation of miR-141 and miR-200 as well as the upregulation of miR-21. In contrast, miR-31 was found to be either down- or upregulated in TAFs depending on the cancer type, revealing that miRNA dysregulation exhibits both common as well as cancer-type specific features [45]. 

The detailed knowledge of miRNAs in lung TAFs is somewhat limited compared to other cancer types [45]. Using a miRNA array, it was observed that 15 miRNAs were differentially expressed in lung TAFs compared to paired CFs, and that miR-101 was the most downregulated miRNA in these TAFs. This study also reported that overexpression of miR-101 abrogated the ability of lung TAFs to enhance cancer cell proliferation and invasion [46]. A more recent miRNA microarray study reported that miR-1 and miR-206 were downregulated, whereas miR-31 was upregulated in lung TAFs compared to paired CFs [47], and forcing these 3 miRNA alterations in CFs was sufficient to induce a TAF-like phenotype in terms of their secretome and tumor-promoting traits in vitro and in vivo [47]. On the other hand, a biomarker study revealed that miR-21 expression in TAFs was associated with poor prognosis in lung ADC [48]. Overall, these studies unveil a growing list of key miRNAs in the aberrant phenotypes of lung TAFs.

## 3. Epigenetics of TAF Activation: Cause or Consequence? 

TAFs are overwhelmingly activated in lung cancer, as illustrated by the positive staining for the standard myofibroblast/activation marker alpha-smooth muscle actin (α-SMA) in virtually all TAFs observed in histologic sections (Figure 1E) [20,31]. Likewise, activated TAFs are found in various proportions in other solid tumors and are critical contributors to tumor-progression [6,7]. Fibroblast activation is elicited by several cytokines, being TGF-β1 the most potent activator known to date [49]. Moreover, there is growing evidence that an abnormally stiff microenvironment, which is a hallmark of NSCLC and other solid tumors, may further contribute to fibroblast activation [50,51]. TGF-β is overexpressed in NSCLC and other solid tumors, and is largely expressed by cancer cells, macrophages and fibroblasts once activated [52,53]. Notably, it is increasingly recognized that epigenetic modifications control fibroblast activation elicited by TGF-β or other cytokines in physiopathological conditions including cancer [10,54] as summarized below. 

### 3.1. Global Hypomethylation and Selective Hypermethylation in Fibroblast Activation

**Figure 2:**
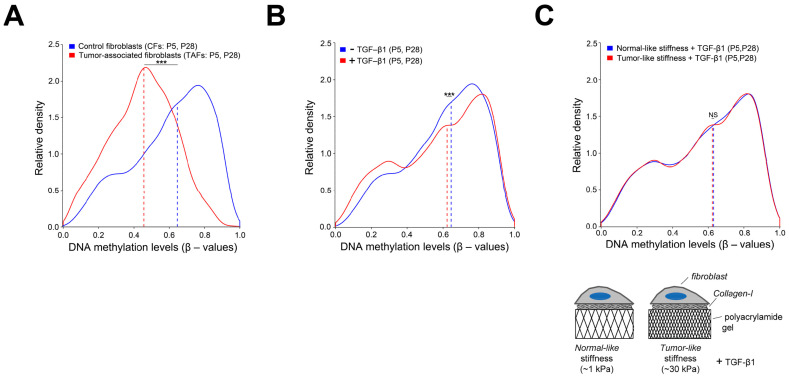
Potential causes of the global DNA hypomethylation of lung TAFs. (**A**) Histogram of the relative density of the average DNA methylation levels (β-values) of 2 TAFs (red line) and paired CFs (blue line). Dashed lines indicate the median β-value of each histogram. *** *p* < 0.001. (**B**,**C**) Histograms of the relative density of the average β-values of 2 CFs cultured either with TGF-β1 in stiff substrata (**B**) or in hydrogels exhibiting soft or stiff (tumor-like) rigidities in the absence of TGF-β1 (**C**). Note that the shift of the median β-value towards lower values elicited by TGF-β1 is larger than that elicited by stiff versus soft hydrogels, yet it did not attain the larger shift observed in TAFs. All plots reprinted from [32] with permission. *** *p* < 0.01.

There is increasing evidence that global hypomethylation may be a common mechanism for myofibroblastic state activation in liver fibrosis [55], supporting that chronic fibroblast activation and DNA hypomethylation may be mechanistically related [56,57,58]. In support of this interpretation, we previously reported that long-term treatment of normal pulmonary fibroblasts with TGF-β1 was sufficient to decrease global DNA methylation (Figure 2A,B), supporting that TGF-β may be a key contributor to global hypomethylation [32]. In this study, we also reported the important TGF-β transcription factor *SMAD3* as the top transcriptionally repressed gene through DNA hypermethylation in lung TAFs, suggesting that TGF-β plays a major role in the epigenetic reprogramming of TAFs. 

Hypermethylation of single genes is also associated with fibroblast activation and fibrosis, including Thy-1/CD90 and T-Box (TBX) transcription factors. Thy-1/CD90 is a common marker for mesenchymal stem cells that is also found in a subset of fibroblasts in an inverse relation with activation markers [59,60]. Thy-1 is chronically lost in pulmonary fibrosis and other fibrotic disorders by epigenetic silencing through the hypermethylation of the promoter region of its coding gene *TYHY1*, and such repression is considered a major driving process of pulmonary fibrosis [59,60]. However, we did not find differential DNA methylation of *TYHY1* in lung TAFs compared to patient-matched CFs [32], suggesting that the link between fibroblast activation and the epigenetic regulation of *TYHY1* may be disease specific. In a separate study, it was reported that treating pulmonary fibroblasts with TGF-β1 downregulated TBX2, TBX4 and TBX5, supporting a potential link between TGF-β1-dependent fibroblast activation and epigenetic repression of these TBX genes, which was reported in lung TAFs [33]. In agreement with this interpretation, we found that these TBX genes were markedly hypermethylated in lung TAFs compared with CFs under basal conditions in culture (i.e., without exogenous TGF-β1), yet such hypermethylation was found within the gene body rather than promoter regions, thereby making its potential transcriptional impact uncertain. Moreover, we did not find an increase in the DNA methylation of these genes upon TGF-β1 stimulation in CFs [32], underscoring that further research is needed to elucidate the transcriptional epigenetic control of TBX genes by TGF-β1 in TAFs.

On the other hand, it was reported that the DNA-binding protein MeCP2 promotes α-SMA expression in pulmonary fibroblasts through direct binding to 3 regions within the promoter of its coding gene *ACTA2*, particularly when it is methylated [61,62]. Conversely, forced MeCP2 downregulation markedly reduced pulmonary fibrosis and fibroblast activation in an in vivo model [61]. Intriguingly, we found that the 3 MeCP2 binding regions within the *ACTA2* promoter exhibited comparable hypermethylation in both lung TAFs and patient-matched CFs, yet α-SMA is overexpressed in TAFs compared to control fibroblasts [31,32], underscoring that elucidating the pro-fibrotic role of MeCP2 in TAFs awaits future investigations.

### 3.2. Histone Core Modifications and DNMTs in Fibroblast Activation 

TGF-β regulates gene expression through *SMAD*-dependent and independent signaling pathways [52]. Notably, the binding of *SMADs* to their target promoters during fibroblast activation was found to be regulated by the methylation of H3 lysine 4 (H3K4), and was in turn dependent on the transcription factor MRTF-A, since silencing MRTF-A reduced the deposition of H3K4 methylation marks on pro-fibrotic promoters and impaired overall TGF-β responses [63,64]. These results underscore that TGF-β/MRTF-A-dependent H3K4 methylation marks on pro-fibrotic gene promoters are major epigenetic regulatory events of fibroblast activation.

There is also evidence that TGF-β1 regulates fibroblast activation by modulating the activity of the transcription factor Snail1. Of note, it was recently reported that TGF-β1/Snail1 enhances the activation of both pulmonary fibroblasts and breast cancer TAFs by cooperating with two protein methyltransferases (PRMT1 and PRMT4) to promote arginine methylation in histones, which drive the expression of fibronectin and other pro-fibrotic genes [65], thereby supporting that histone methylation is involved in fibroblast activation and fibrosis [66]. However, the role of PRMTs in lung TAFs remains unexplored.

In addition to PRMTs, HDACs also are involved in fibroblast activation [66]. HDAC4 appears to regulate the *SMAD*-independent Akt signaling downstream of TGF-β1 [67], and genetical depletion of HDAC4, 6 and 8 prevented TGF-β-induced fibroblast activation, whereas forcing the overexpression of selected DNMTs in lung fibroblasts acted otherwise [68]. Similarly, a selective inhibitor of HDACs 1, 3 and 8 impaired the basal and TGF-β-induced activation of murine TAFs and breast cancer TAFs [10], supporting that altered histones may be a key process underlying the aberrant chronification of activated fibroblasts as observed in the desmoplastic tumor stroma.

On the other hand, TGF-β1 can upregulate NNMT [69], and a recent proteomic study in ovarian TAFs reported that NNMT was upregulated and its expression was necessary and sufficient to elicit hallmarks of activated TAFs, including expression of α-SMA and pro-fibrotic ECM components as well as enhanced contractility, concomitantly with the acquisition of a tumor-promoting phenotype. Conversely, inhibition of NNMT activity with shRNA (shNNMT) both reverted the expression of markers of activated TAFs and attenuated their tumor-promoting traits. Remarkably, the activation and tumor-promoting traits of TAFs were rescued by inhibition of H3K27 trimethylation with the EZH2 histone methyltransferase inhibitor DZNep as well as the general methyltransferase inhibitor 3-DZA [37], further underscoring the role of altered histones in TAF activation.

### 3.3. MiRNAs in Fibroblast Activation 

Numerous miRNAs have been associated with the acquisition of a desmoplastic stroma rich in activated TAFs. Likewise, different miRNAs have been implicated with myofibroblast “memory”, and many of them are directly regulated by TGF-β [45,66]. In breast cancer it was found that downregulation of miR-29 in TAFs promoted cancer cell growth and metastasis [70]. In contrast, overexpression of miR-29 suppressed fibrosis in different organs, and similar suppressive roles were reported for miR-200a [66]. In the context of the lung, TGF-β downregulated miR-29, whereas forcing miR-29 downregulation in lung fibroblasts was sufficient to upregulate the expression of pro-fibrotic genes [71]. On the other hand, it was reported that PPAR-γ is a natural repressor of α-SMA that is negatively regulated by MeCP2. As an example of the interplay between miRNAs and other epigenetic regulatory processes in fibroblast activation, it was reported that the downregulation of miRNA-132 elicited higher levels of MeCP2 expression, which recruits HP-1 transcriptional repressor into the PPAR-γ promoter region and induces loss of expression through the enhanced methylation of H3K9 and H3K27 of the PPAR-γ gene in myofibroblasts [72]. In line with these findings, studies based on miRNA profiling revealed how the dysregulation of some miRNAs can modulate the TGF-β pathway in TAFs of breast and prostate cancers [73,74]. In addition, there is evidence that TGF-β1 regulates itself through miRNAs, since the activity of the *TGFB1* promoter was upregulated by TGF-β1 and miR-192 in mouse mesangial cells [75]. 

In lung ADC, miR-21 expression was reported in both cancer cells and TAFs, and upregulating miR-21 in lung fibroblasts was sufficient to promote activation markers such as periostin, α-SMA and podoplanin as well as to induce the novel TAF-secreted protein calumenin [48]. Likewise, miR-21 has been implicated in TAF activation in numerous cancer types, supporting that miR-21 is a prominent driver of TAF activation and consequent tumor promotion [45,48]. However, we still lack a complete picture of how miRNAs drive the aberrant activation of TAFs in NSCLC and other solid tumors. Similarly, a deeper characterization of the role of DNA methylation and histone modifications in the aberrant activation of lung TAFs is required.

## 4. Emerging Mechanisms Underlying the Epigenetic Reprogramming of Lung TAFs

The ultimate causes of the epigenetic reprogramming of lung TAFs remain to be determined, and are likely to be heterogeneous and even patient-type dependent. However, some key emerging mechanisms have been identified in recent years that are summarized below.

### 4.1. Crosstalk with Cancer Cells 

Most solid tumors, including major lung cancer subtypes, take years to develop, thereby enabling long-term paracrine interactions between cancer cells and surrounding stromal cells including TAFs [76]. Such long interaction is assumed to drive the co-evolution of both cancer cells and TAFs, including epigenetic changes in both cell populations that, in some patients, may be key for tumor progression. Likewise, epigenetic reprogramming may arise at sites of chronic inflammation and fibrosis [6]. As an example of epigenetic reprogramming of TAFs by cancer cells, it was reported that TAFs can become epigenetically activated by cytokines secreted by tumor cells such as the leukemia inducible factor LIF, which induced methylation of SHP1 and subsequently the activation of JAK1/STAT3 signaling concomitantly with the alteration of the contractile cytoskeleton and histone acetylation. Notably, all these changes promoted fibroblast activation further as well as the acquisition of a tumor-promoting phenotype in TAFs derived from lung as well as head and neck and breast human carcinomas [77]. In the context of the lung it also was reported that miR-101 is the most downregulated miRNA in lung TAFs compared to paired CFs, and that treating CFs with conditioned medium from a panel of lung cancer cell lines was sufficient to reduce miR-101, implicating a soluble heterotypic crosstalk between cancer cells and fibroblasts in such downregulation [46].

A major mechanism of paracrine heterotypic cell communication that is drawing increasing attention is based on the secretion of exosomes and other extracellular vesicles. Of note, a recent microarray study comparing the exosomal content of high- and low-metastatic cancer cells reported that high-metastatic hepatocellular carcinoma (HCC) cells have a greater capacity to convert normal fibroblasts into TAFs through the secretion of exosomal miR-1247-3p, which directly targets B4GALT3, leading to the activation of β1-integrin/NF-κB signaling in fibroblasts. Activated TAFs in turn promoted cancer progression by secreting pro-inflammatory cytokines, including IL-6 and -8. Moreover, high levels of exosomal miR-1247-3p were associated with lung metastasis in HCC patients (Fang et al., 2018). In lung cancer, miR-369 overexpression was reported in extracellular vesicles in TAFs, which promoted lung cancer cell growth, migration, invasion and tumorigenesis [78]. 

### 4.2. Extracellular and Intracellular Mechanical Cues 

Lungs are soft and elastic to support the cyclic volume changes required for normal breathing. Likewise, normal tissue elasticity supports physiologic tissue-specific functions in other organs. Fibroblasts are the key cell type in controlling tissue elasticity through their own contractility and through their ability to deposit ECM and remodel it [60]. Notably, a hallmark of NSCLC and other solid tumors is a dramatic increase in tissue rigidity that has been largely associated with the desmoplastic stroma rich in hypercontractile/activated TAFs in the background of an excessive deposition of fibrillar collagens [32,66]. Similar mechanical stiffening is also a hallmark of organ fibrosis, and is increasingly regarded as a major driving force of both tumor progression and fibrosis expansion [20,66]. Interestingly, there are growing links between both extracellular and intracellular mechanical cues and epigenetic alterations in fibroblasts [63]. 

By culturing pulmonary fibroblasts on hydrogels with tunable elasticity spanning normal-like and fibrotic-like conditions, it was observed that fibroblast activation was low in soft hydrogels, whereas it increased in stiff hydrogels. Of note, fibroblasts cultured for 3 weeks on stiff (pathologic-like) hydrogels and subsequently seeded on soft (normal-like) hydrogels retained myofibroblastic features up to 2 weeks. Conversely, culturing fibroblasts on soft hydrogels for 3 weeks partially protected them for activation when seeded on stiff hydrogels [79]. This elegant in vitro study provided the first direct evidence of “mechanical memory” in lung fibroblasts. To assess whether such “mechanical memory” was caused by changes in DNA methylation, we cultured pulmonary fibroblasts for 5 days on stiff hydrogels in the presence of TGF-β1, but found a modest reduction in global DNA methylation that did not reach statistical significance (Figure 2C) [32]. Moreover, these methylation changes involved only 19 CpG sites, but none within promoter regions, supporting that the “mechanical memory” in fibroblasts may be driven by mechanisms other than DNA methylation alterations. In contrast, other studies reported that MRTF-A and miR-21 may play an important role in the “mechanical memory” of fibroblasts. Thus, miR-21 is an important transcriptional regulator of pro-fibrotic genes in fibroblasts, and its expression was upregulated in mesenchymal stem cells cultured for several passages on stiff substrata concomitantly with the acquisition of a pro-fibrotic phenotype, which persisted even when cultured on soft substrata. On the other hand, MRTF-A promoted miR-21 transcription in cells cultured on stiff rather than soft substrata, suggesting that MRTF-A plays a key role in transducing extracellular mechanical cues into epigenetic alterations in fibroblasts and other mesenchymal cells towards the acquisition of a persistent pro-fibrotic phenotype [80,81]. 

In addition to miRNAs, biochemical and biomechanical cues from the local ECM can alter cell shape and mechanics by remodeling the actin cytoskeleton [82], which has been identified as an important epigenetic regulator through changes in histone acetylation and chromatin organization [63,83]. Specifically, an increase in cell spreading and cytoskeletal tension have been linked to an increase in H3 and H4 acetylation, which contributes to the formation of transcriptionally active chromatin [63,83]. The underlying mechanisms may involve the actin cytoskeleton through at least two complementary processes. First, actin filaments may sequester HDACs, which may directly modulate gene expression by altering the nuclear translocation of HDACs [63,84]. Secondly, an increase in actin polymerization may unleash MRTF-A, for it is sequestered by non-polymerized globular actin [85]. Collectively, these observations support that ECM stiffening and subsequent changes in the cytoskeleton and cell shape may alter the epigenetic landscape of fibroblasts to synergize with biochemical cues such as TGF-β to promote or even perpetuate the acquisition of an activated/pro-fibrotic phenotype. It remains to be determined whether a similar mechanical epigenetic reprogramming occurs in TAFs.

### 4.3. Smoking and Other Environmental Factors 

Smoking is a major risk factor for lung cancer and other cancer types, and it is associated with diverse epigenetic alterations. Thus, several epigenome-wide screening studies have reported a marked impact of cigarette smoke on global DNA methylation, including the reduction of the methylation levels of CpG island neighbor regions (called shores) across the genome in a similar fashion as aging [86,87], supporting the interpretation of the biological impact of tobacco smoking as accelerated aging. Tobacco smoking further modulates the epigenomic landscape at different levels. First, tobacco constituents such as nicotine may alter the levels of selected DNMTs, including the downregulation of DNMT3A and DNMT3B [88] and the upregulation of DNMT1 [89]. Second, carcinogens in cigarette smoke such as nitrosamines or formaldehyde can damage DNA by introducing double strand breaks, which is a main cause of mutations. As a consequence, the DNA repair machinery recruits DNMT1 to the double strand breaks, where it may induce the aberrant methylation of adjacent sites [90]. Similarly, nicotine-derived nitrosamine ketone is a potent cigarette carcinogen that can attenuate DNMT1 degradation and increase its nuclear accumulation further, which has been associated with the downregulation of tumor suppressor genes in NSCLC through DNA methylation [91]. Third, cigarette smoke condensate increases the expression and presence of Sp1 transcription factor, which has high binding affinity to CpG sites. This has a negative effect on global DNA methylation because the aberrant overloaded deposition of Sp1 across the genome may prevent many CpG sites from keeping the normal methylation patterns [92]. 

**Figure 3:**
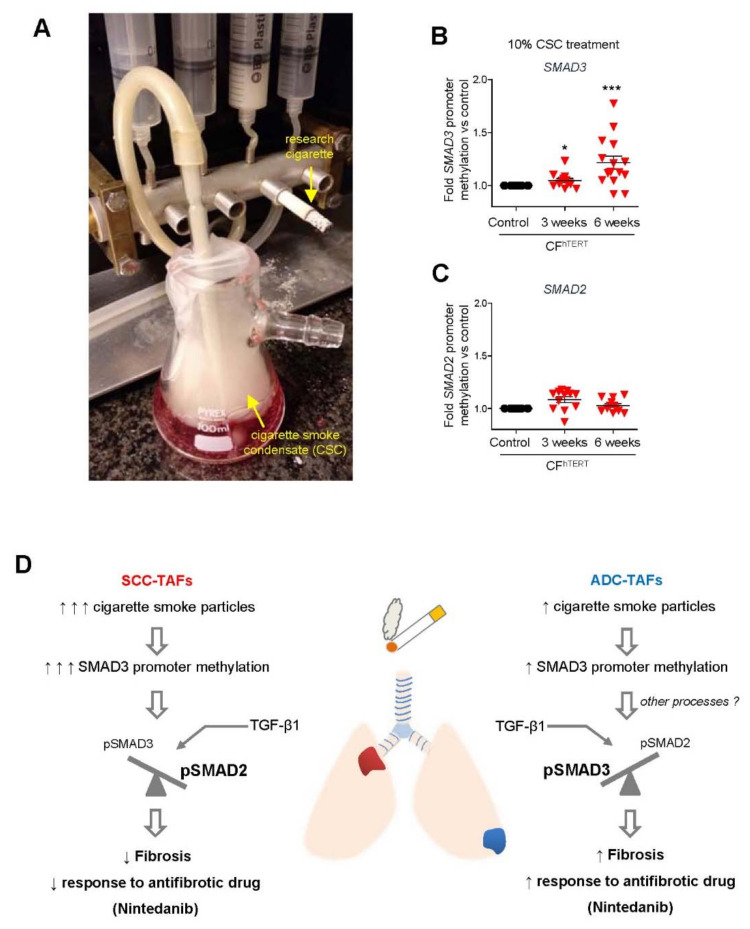
Smoking and *SMAD3* epigenetic repression in lung TAFs. (**A**) Picture of the set-up used to obtain cigarette smoke condensate (CSC) to stimulate CFs. (**B**,**C**) Fold *SMAD3* (**B**) and *SMAD2* (**C**) Promoter DNA methylation of CFs stimulated with 10% CSC up to 6 weeks assessed by pyrosequencing, revealing that smoking particles are sufficient to increase selectively promoter hypermethylation in *SMAD3* but not its closely related homolog *SMAD2*. * *p* < 0.05; *** *p* < 0.005. (**D**) Outline of the emerging model illustrating the differential relationship between smoking, the anatomic location, *SMAD3* hypermethylation in TAFs and their response to the antifibrotic drug Nintedanib in ADC compared to SCC. (**B**–**D**) Reprinted from [19] with permission.

In agreement with all the evidence supporting a major epigenetic impact of smoking, we found a global hypomethylation in lung TAFs derived from current smokers [32]. Yet we also found that *SMAD3* was transcriptionally repressed through promoter hypermethylation, particularly in SCC (Figure 1D) [19,32], which is a lung cancer subtype strongly associated with smoking. Notably, we also reported that stimulating normal pulmonary fibroblasts with cigarette smoke condensate in culture was sufficient to time- and dose-dependently increase the promoter methylation of *SMAD3* but not that of its closely related homolog *SMAD2* [19], as summarized in Figure 3A–C. Although the underlying mechanisms of the selective *SMAD3* hypermethylation remains unclear, it is conceivable that the common DNMT1 increase in smokers may be involved. Intriguingly, it was also reported that cigarette smoke condensate in culture induced the transcriptional repression of *SMAD3* in lung cancer cell lines through histone deacetylation rather than DNA methylation [93], supporting that smoking represses selectively *SMAD3* through distinct epigenetic processes depending on the cell type. 

Our observed larger *SMAD3* epigenetic repression in TAFs from lung SCC compared to ADC patients has translational consequences. First, it suggests that fibrosis should be larger in ADC compared to SCC, which we confirmed recently in lung TAFs and in patient samples [19]. Second, it provides a rationale for the puzzling epidemiologic observation that smoking is associated with lower risk of radiotherapy-induced pneumonitis and ultimately fibrosis [94], which is a major unwanted side effect of radiotherapy that poses a serious limitation to its efficacy in cancer.

### 4.4. Hypoxia

A common hallmark of aggressive tumors is the appearance of hypoxic regions in tumors that grow at a faster rate than their ability to become vascularized [95]. Hypoxia was reported to induce global hypomethylation in colorectal and melanoma cells in both fibroblasts and cancer cells [96,97]. Consistently, an inverse relationship between tumor hypoxia and methylation was observed in tumor xenografts [96]. In contrast, global hypermethylation including the epigenetic repression of *TYHY1* was reported in hypoxic pulmonary fibroblasts compared to normoxic conditions concomitantly with increased expression of α-SMA and other activation markers [98]. These apparently contradictory observations reveal that clarifying the role of hypoxia in global DNA hypomethylation and TAF activation requires further investigations.

## 5. Potential Therapeutic Implications

The development of targeted therapies against driver mutations and, more recently, of immunotherapy has led to unprecedented survival benefits in NSCLC. However, acquired resistance to targeted therapies is common, and current positive responses to immunotherapy are restricted to selected patients. Consequently, the overall cure and survival rates for NSCLC remain low, underlying the need to develop more efficient therapeutic strategies [99]. In this context, a promising emerging approach is based on combinatorial therapies that target both TAFs and cancer cells [9]. 

Regarding TAF-specific therapeutics, it is expected that an improved understanding of the epigenetic regulation of TAFs may facilitate the identification of efficient therapeutic strategies against their tumor-promoting epigenetic reprogramming. On the other hand, there is mounting evidence that non-activated TAFs do not contribute to tumor progression [7,100], supporting a therapeutic strategy aiming to epigenetically reprogram TAFs towards a non-activated phenotype or even a tumor-restraining phenotype [23,101]. Accordingly, it is unsurprising that identifying therapeutic approaches to target activated TAFs such as inhibitors of pro-fibrotic cytokines or aberrant mechanical signaling is drawing increasing interest. These therapeutic options were reviewed in detail elsewhere [23,102]. Here we will focus on the limited yet expanding therapeutic strategies based on the epigenetic changes of TAFs, including antifibrotic drugs and other drug types.

### 5.1. Limitations of Antifibrotic Drugs in Lung Cancer: Unexpected Epigenetic Influence of Smoking 

A promising approach in cancer therapeutics is based on combining anti-cancer drugs with anti-stromal drugs targeting tumor fibrosis or other aberrant aspects of the desmoplastic tumor stroma [76,103]. Our observed larger tumor fibrosis in TAFs and patient samples from ADC compared to SCC strongly suggests that ADC-TAFs could be more responsive to antifibrotic therapies than SCC-TAFs. In support of this interpretation, we recently showed that the selective therapeutic benefits of the antifibrotic drug nintedanib reported in ADC in the LUME-1 clinical trial [22] (Table 1) could be reproduced in vitro using co-cultures of cancer cells and TAFs, since nintedanib abrogated both the extent of activation/fibrosis in TAFs as well as the pro-growth and pro-invasion effects of TAFs’ conditioned medium in cancer cells in ADC but not SCC [21]. We subsequently showed that the larger epigenetic repression of the important pro-fibrotic transcription factor *SMAD3* in SCC-TAFs compared to ADC-TAFs elicited a compensatory increase in the activity of its closely related homolog *SMAD2*, which is not pro-fibrotic, and how this epigenetic control of the *SMAD3*/*SMAD2* balance is a key process underlying the lack of therapeutic effects of nintedanib in SCC in vitro and in vivo [19]. Furthermore, both *SMAD2* and *SMAD3* are activated by phosphorylation, and our results support that patients whose TAFs exhibit a low (<1) *pSMAD3*/*pSMAD2* ratio may be refractory to nintedanib and possibly to other antifibrotic drugs, whereas those with high (>1) *pSMAD3*/*pSMAD2* ratio may elicit positive responses (where *pSMAD2* and *pSMAD3* refers to phosphorylated *SMAD2* and *SMAD3*, respectively) (Figure 3D). Finally, our results support that nintedanib (and possibly other antifibrotic drugs) may be particularly useful in overcoming radiotherapy-induced fibrosis in ADC, which has been pointed to as a major process underling pulmonary toxicity and ultimately resistance to radiotherapy [104]. 

### 5.2. Drugs That Alter Histone Marks in Fibroblasts

Scriptaid, discovered in a high-throughput screening as a deacetylase inhibitor [113], has shown therapeutic effects against activated TAFs. Specifically, Scriptaid inhibited HDAC 1/3/8 and downregulated several activation markers of TAFs from either human breast tumors or murine melanomas [10]. Moreover, ECM deposited by Scriptaid-treated murine melanoma TAFs decreased cancer cell spreading and attachment compared to controls, and administration of Scriptaid inhibited tumor growth and decreased fibroblast activation in vivo [10]. However, sustained treatment with Scriptaid was needed to keep TAFs in a deactivated state, revealing that this drug cannot induce a stable epigenetic reprogramming. In addition, it cannot be ruled out that Scriptaid acts on additional HDACs or even non-histone substrates. Although Scriptaid has not been tested in clinical settings yet, its derivative SAHA (also known as Vorinostat) has shown anticancer effects in clinical studies [114] (Table 1), and has downregulated activation markers in fibroblasts from patients with idiopathic pulmonary fibrosis and in murine models of pulmonary fibrosis [115], although its performance in TAFs has not been determined. In NSCLC, Vorinostat has been used in clinical trials with other agents, including carboplatin and paclitaxel [110] and the PD-1 inhibitor Pembrolizumab [109], which have reported enhanced therapeutic effects and increased patient survival. Similar positive results have been reported in combination clinical studies in other cancer types, supporting the use of Vorinostat in combinational therapies rather than monotherapy. However, studies of HDAC inhibitors (particularly HDAC 2) in solid tumors have reported that they may increase the expression of common factors of the senescence-associated secretory phenotype or SASP that may be tumor-promoting [10]. Therefore, further studies are required to elucidate the global therapeutic potential of HDAC inhibitors against TAFs in solid tumors. 

### 5.3. Drugs That Modify DNA Methylation Marks in Fibroblasts and Mesenchymal Cells

A hallmark of the desmoplastic stroma in NSCLC and other solid tumors is an excessive deposition of fibrillar collagens. Collagen biosynthesis is regulated by several post-transcriptional modifications, in which collagen prolyl hydroxylation (CPH) is the most prevalent in humans and is catalyzed by P4H proteins that require vitamin C as a cofactor [116]. Intriguingly, a recent study reported that an increase in CPH in pluripotent stem cells and breast cancer cells elicited an increase in collagen synthesis concomitantly with a reduced activity of the demethylases TET and JMJ, which require also vitamin C as a cofactor, which in turn yielded an increase in global DNA/histone hydroxylation and H3K9 methylation [105]. Notably, these epigenetic effects enhanced epithelial-mesenchymal transition (EMT) in breast cancer cells and metastasis in in vivo models. Moreover, a drug screening analysis identified the anti-asthmatic agent budesonide as an effective inhibitor of P4H activity and subsequent downregulation of CPH, collagen deposition as well as global DNA/histone methylation through enhanced activity of TET/JMJ demethylases, which altogether reduced EMT and metastasis in breast cancer cells [105] (Table 1). This work identified CPH as a novel epigenetic modulator of epithelial plasticity through its antagonistic effects on DNA/histone hydroxylation driven by the available vitamin C, which may be relevant in TAFs because they rely also on CPH to elicit the excessive collagen deposition within the desmoplastic tumor stroma. In support of this interpretation, it was reported that budesonide prevents pulmonary fibrosis in vivo [117]. In contrast, the therapeutic use of budesonide in TAFs is currently unknown. Yet, Budesonide was examined as a monotherapy in a phase II clinical trial in patients with high risk of developing lung cancer, in which it did not demonstrate decreased lung nodules compared to control groups [118], suggesting that further studies are required to elucidate the potential therapeutic use of this drug either as monotherapy or as combinatorial therapy in cancer. 

On the other hand, following the recent discovery that TGF-β1/Snail1 elicits histone modifications in fibroblast by cooperating with two protein methyltransferases, inhibitors of methyltransferases were tested, including sinefungin and arginine methyltransferase inhibitor 1 (AMI-1) (Table 1), and were found to prevent myofibroblast activity in culture and in vivo, as well as to reduce metastasis in a mouse breast cancer model [65]. These results support that methyltransferase inhibitors hold potential against the tumor-promoting effects of activated TAFs [65]. Similarly, it was reported that treating hepatic stellate cells with the global DNA demethylating agent 5-AZA prevented their activation towards a myofibroblast-like phenotype [119]. However, preliminary results from our group could not find a robust inhibition of TGF-β-induced activation of lung TAFs in the presence of 5-AZA in culture (unpublished results), suggesting that the translational potential of this drug to epigenetically reprogram TAFs remains uncertain.

### 5.4. Drugs of Nuclear Vitamin D Receptor

There is solid evidence that vitamin D metabolites or analogs may act as effective inhibitors of TAFs or TAF-like cells such as stellate cells. The vitamin D analogue calcipotriol decreased the proliferation and the release of tumor-promoting factors in pancreatic TAFs [107], and similar results were obtained with vitamin D supplementation in vitro [120] (Table 1). Likewise, treating activated stellate cells with a nuclear vitamin D receptor (VDR) ligand was sufficient to reprogram them towards a more quiescent phenotype and reduced overall tumor progression [106]. Once activated, VDR may recruit epigenetic coactivators, including HATs and HDACs, eliciting chromatin modifications and subsequently altering gene expression [121]. In colorectal cancer, it was reported that the VDR natural ligand 1,25-dihydroxyvitamin D3 inhibited protumoral activation of TAFs, eliciting a gene transcriptional program associated with increased survival (reviewed in [122]). In contrast, our current understanding of the epigenetic effects of vitamin D and VDR on lung TAFs remains limited. Likewise, even though there are several clinical trials examining vitamin D in cancer, very few have been conducted in lung cancer, with the exception of a phase I/II trial combining calcitriol (active form of vitamin D) with docetaxel and cisplatin. This trial did not demonstrate decreased tumor growth compared to control groups treated with chemotherapy; however, an association between disease progression and some SNPs of the regulator of the vitamin D degradation enzyme coded by *CYP24A1* gene was reported, suggesting that patient stratification based on selected *CYP24A1* SNPs should be considered in future studies with vitamin D-related compounds [108].

## 6. Conclusions

TAFs are involved in all steps of tumor progression and even in resistance to therapies in NSCLC and other solid tumors. There is growing evidence that TAFs are epigenetically reprogrammed, as illustrated by the observation of global DNA hypomethylation concomitantly with hypermethylation of selective genes such as *SMAD3*, histone core modifications driven by N-methyltransferases, and the dysregulation of numerous miRNAs. Likewise, most TAFs exhibit an activated/myofibroblast-like phenotype, which is driven and/or maintained by epigenetic modifications that may synergyze with or modulate the potent fibroblast activator cytokine TGF-β1. Although the mechanisms underlying the persistent activation and epigenetic reprogramming of TAFs remain incompletely understood, some key processes were recently identified, including the paracrine crosstalk with cancer cells, aberrant mechanical cues elicited by either the abnormally stiff ECM or the actomyosin cytoskeleton, and unwanted effects of cigarette smoke particles. Indeed, smoking particles alone can induce the epigenetic repression of *SMAD3* in normal pulmonary fibroblasts through increased hypermethylation, which may limit the response to antifibrotic drugs, particularly in lung cancer types strongly associated with smoking such as squamous cell carcinoma. Finally, it is worth noting that our increasing understanding of the epigenetic reprogramming of TAFs supports novel therapeutic strategies aiming to re-reprogram them towards a non-activated or even a tumor-restraining phenotype. Promising therapeutic strategies include drugs that target epigenetic regulators directly (i.e., HDAC or methyltransferase inhibitors) or indirectly, such as vitamin D analogues or budesonide. 

## Figures and Tables

**Table 1:** List of major drugs that benefit from the aberrant epigenetic reprogramming of TAFs.

Major Known Targets in Fibroblasts	Drug	Cancer Type	Clinical Status	Reference
protein arginine methyltransferases (PRMTs)	arginine methyltransferase inhibitor 1 (AMI-1), sinefungin	Breast	in vivo preclinical study	[65]
Collagen prolyl hydroxylation	Budesonide	Breast	Clinically approved to treat asthma	[105]
Nuclear Vitamin D receptor	Calcipotriol, paricalcitol	Pancreas, colorectal cancer, NSCLC	Preclinical or Phase I/II	[106,107,108]
VEGFRs, PDGFRs, FGFRs, TGF-β1/*SMAD3*	Nintedanib	NSCLC (ADC)	Clinically approved to treat advanced ADC	[19,21,22]
Histone deacetylases (HDACs)	Vorinostat	NSCLC, kidney, myeloma	Clinically approved for T-cell lymphoma	[109,110,111,112]
